# HIV-Associated CD8 Encephalitis: A UK Case Series and Review of Histopathologically Confirmed Cases

**DOI:** 10.3389/fneur.2021.628296

**Published:** 2021-04-01

**Authors:** Sebastian B. Lucas, Kum T. Wong, Sam Nightingale, Robert F. Miller

**Affiliations:** ^1^Department of Cellular Pathology, Guys and St. Thomas' NHS Foundation Trust, London, United Kingdom; ^2^Department of Pathology, Faculty of Medicine, University of Malaya, Kuala Lumpur, Malaysia; ^3^Department of Neurology, University of Cape Town, Cape Town, South Africa; ^4^Centre for Clinical Research in Infection and Sexual Health, Institute for Global Health, University College London, London, United Kingdom; ^5^Clinical Research Department, Faculty of Infectious and Tropical Diseases, London School of Hygiene and Tropical Medicine, London, United Kingdom; ^6^Mortimer Market Centre, Central and North West London NHS Foundation Trust, London, United Kingdom

**Keywords:** HIV, autopsy, brain, CD8 encephalitis, antiretroviral therapy, viral escape, corticosteroids, immune reconstitution inflammatory syndrome

## Abstract

HIV-associated CD8-encephalitis (HIV-CD8E) is a severe inflammatory disorder dominated by infiltration of the brain by CD8^+^ T-lymphocytes. It occurs in people with HIV, typically when the virus is apparently well-controlled by antiretroviral treatment (ART). HIV-CD8E presents with symptoms and signs related to marked cerebral inflammation and swelling, and can lead to coma and death unless treated promptly with corticosteroids. Risk events such as intercurrent infection, antiretroviral therapy interruption, immune reconstitution inflammatory syndrome (IRIS) after starting ART, and concomitant associations such as cerebrospinal fluid (CSF) HIV viral escape have been identified, but the pathogenesis of the disorder is not known. We present the largest case series of HIV-CD8E to date (*n* = 23), representing histopathologically confirmed cases in the UK. We also summarize the global literature representing all previously published cases with histopathological confirmation (*n* = 30). A new variant of HIV-CD8E is described, occurring on a background of HIV encephalitis (HIVE).Together these series, totalling 53 patients, provide new insights. CSF HIV viral escape was a frequent finding in HIV-CD8E occurring in 68% of those with CSF available and tested; ART interruption and IRIS were important, both occurring in 27%. Black ethnicity appeared to be a key risk factor; all but two UK cases were African, as were the majority of the previously published cases in which ethnicity was stated. We discuss potential pathogenic mechanisms, but there is no unifying explanation over all the HIV-CD8E scenarios.

## Introduction

In 2002, we [SL, RM] documented the first case of what is now termed HIV-associated CD8 encephalitis (HIV-CD8E)—an inflammatory cerebral disease dominated by severe infiltration of the cerebrum by CD8+ T-cells (1). Subsequently, several authors have documented further cases of HIV-CD8E. While at first most, if not all, patients with HIV-CD8E died (the literature does not provide precise case date details), many now survive with administration of corticosteroid therapy, with or without modification of antiretroviral treatment (ART).

CD8^+^ T-cell infiltration of the brain is not unique to HIV infection, occurring in many other encephalitis syndromes (infectious, autoimmune, and idiopathic), where diffuse and perivascular CD8^+^ T-cell infiltrates are seen histopathologically in the brain (2). Over two decades, this syndrome has come to be recognized by clinicians as an uncommon complication occurring in some patients being treated with ART. Clinically, HIV-CD8E presents as an acute or subacute deterioration of cerebral function often with progression to coma and death if not arrested. Imaging shows diffuse white matter changes and cerebral swelling/oedema. Definitive diagnosis is made on brain biopsy or at autopsy, where diffuse, predominantly white matter, infiltration by CD8^+^ T-cells is seen (normal brains do not have T-cells visible in the parenchyma, only within the vasculature). The main differential diagnoses—opportunistic infections, primary vasculitis, and lymphoma—are excluded through histopathological, microbiological and DNA analysis of brain tissue and/or cerebrospinal fluid (CSF). HIV-CD8E is clinico-pathologically distinct from classical “HIV encephalitis (HIVE)” where pathology shows HIV-containing microglial cells on immunohistochemistry, microglial nodules, microglial giant cells and few T-cells (3, 4), and which presents with a sub-acute to chronic subcortical dementia syndrome.

Pathogenetically, it is still unclear why CD8^+^ T-cells flood into the brain, and why it swells. Several associations and risk events for HIV-CD8E have been previously identified including: intercurrent extra-cerebral infection or malignancy, CSF virological escape (5, 6), ART treatment interruption, immune reconstitution inflammatory syndrome (IRIS) (1, 7), and antiretroviral drug resistance (8). Occasionally HIV-CD8E occurs in patients who have never been treated with ART (9).

Here we describe a case series which represents to our knowledge the total clinico-pathological experience of HIV-CD8E in the UK to date. We also review all previously published cases with histopathological confirmation in the global literature. The purpose is: first, to present the largest case series of HIV-CD8E patients yet described; second, to summarize the global literature on HIV-CD8E with neuropathology; third, to identify common associations and risk factors; and fourth, to discuss potential pathological mechanisms of how and why HIV-CD8E develops.

## Methods

### Part 1. UK Case Series

We reviewed cases of HIV-CD8E that were confirmed neuropathologically (on biopsy or at autopsy) in the UK between 2002 and February 2021. Cases were identified through the diagnostic experience of SBL, who ran a national consultation service for HIV-related pathology, and the clinical HIV links of RFM, and review of cases presented at HIV conferences and published case reports.

For each case of HIV-CD8E, clinical case records were reviewed and data extracted including, detail of clinical presentation, ART regimen, CD4^+^ and CD8^+^ T-cell counts in blood at time of presentation, and in the period prior to presentation, HIV viral load in plasma at presentation and previously, and results of brain CT and magnetic resonance (MR) imaging, where performed. CSF lymphocyte counts and CSF HIV viral loads were recorded where available. Clinical evidence of intercurrent infection prior to presentation with HIV-CD8E was noted. In patients on ART, the regimes were evaluated with respect to drug type, CNS penetration effectiveness (CPE) score (10), timing of initiation of ART, and any drug resistance. Systematic data on the HIV clades in the patients were not collected.

Pathologically, all autopsied cases had a complete autopsy with all-organ histology. For these and the brain biopsy cases, the brain histopathological standard work-up was:

H&E slide preparations;

CD4, CD3, CD8, CD20 immunohistochemistry (IHC) for lymphocyte typing;

HIVp24 IHC for HIV-1 infection, and

CD68 IHC for microglial cells and macrophages.

Some cases also had the following:

CD38 IHC for activated T-cells;

CD56 IHC for natural killer cells;

SV40 IHC for JC virus;

HSV-1 IHC for herpes simplex virus (HSV);

EBER *in situ* hybridization for Epstein-Barr virus (EBV) infection;

*Toxoplasma gondii* IHC, and

GFAP IHC for astrocytes.

The manufacturer sources of antibodies for IHC studies changed over the near 20 years of the study, but all antibodies were appropriately controlled with positive and negative controls during preparation of stained slides.

Four patients had clonality PCR assays for the β and γ chains of the T-cell receptor performed on formalin-fixed paraffin-embedded (FFPE) brain tissue.

Two patients had standard PCR and reverse transcriptase-PCR assays on fresh autopsy brain tissue to detect common viruses: cytomegalovirus (CMV), JC virus, HSV, varicella zoster virus (VZV), EBV, enterovirus, adenovirus, parvovirus, West Nile virus, human herpes viruses 6 and 8 (HHV6 and HHV8), and HIV-1.

One patient had viral genome extracted from FFPE brain tissue to look for HIV.

Twelve patients (four survivors and eight deceased) had pre-mortem and/or peri-brain biopsy CSF viral studies performed—looking for the same viruses evaluated in autopsy brain tissues. No autopsied cases had autopsy CSF examined.

One patient had autoantibody studies undertaken for autoimmune encephalitis.

### Part 2. Global Published Cases

We performed a literature review of published cases with histopathological confirmation. Search terms were: CD8, encephalitis, encephalopathy, HIV, human immunodeficiency virus, neuropathology, brain biopsy, autopsy, antiretroviral therapy, ART, CSF, viral escape, immune reconstitution, IRIS, corticosteroids. Inclusion criteria were case reports and case series, published in English between January 2000 and 2021: review articles were excluded. Cases were reviewed to determine whether brain biopsy or autopsy was performed: only cases with histopathological confirmation of HIV-CD8E were included.

For each case, data was collected on demographics, clinical presentation, treatment, outcome, HIV viral load (plasma and CSF) and CD4^+^ T-cell count. Risk events were divided into the following categories based on available information: well-controlled HIV infection, i.e., HIV-CD8E occurring without an identified risk event; intercurrent infection or malignancy; ART treatment interruption/poor adherence; IRIS after commencing ART; ART drug resistance; not in receipt of ART; EBV-associated. In some instances the original published risk event was changed following re-interpretation of reported findings.

## Results

### UK Case Series

From 2002-February 2021, we identified, in the UK, 23 adults infected with HIV-1 who had HIV-CD8E, diagnosed at autopsy (*n* = 19) or on brain biopsy (*n* = 4). Thirteen of the autopsy examinations were medico-legal, initiated by Coroners in England to obtain the precise cause of death when the cause was not evident; in six cases, they were hospital/consented autopsies initiated by the treating clinicians. Spinal cords were occasionally removed at autopsy. In most cases, the brains were fixed in formalin prior to blocking. The brains were comprehensively sampled to include supra- and sub-tentorial zones, processed to paraffin-embedding, then cut for histology slides. The autopsies were mostly performed by SBL; some were performed by other pathologists [see Acknowledgments] who consulted on diagnosis.

The four brain biopsies were performed for diagnostic reasons (to confirm or exclude lymphoma or specific infection). The biopsies were all reviewed by SBL.

The pathological diagnosis of HIV-CD8E was based on characteristic brain histopathology: diffuse cerebral infiltration by CD8^+^ T-cells and microglial activation, and the absence of other specific causes of encephalitis.

#### Clinical

Clinical, imaging, and laboratory findings of the 23 patients are summarized in Table 1. (specific patients are indicated with Case #number). Twenty-one patients were of Black African ethnicity, two were white; 15 patients (65%) were female. The median age was 41.5 years (range 19–52) and HIV infection was diagnosed at a median of 9 years (range 0–24) prior to presentation. At presentation with HIV-CD8E, 17 patients (74%) were receiving ART, with a mean CPE score of 6.7 (range 2–12) (10); four patients (17%) had received ART but had stopped it 1–5 months previously, CPE score range = 3–8. Two patients (9%) had never received ART.

**Table 1:** Twenty-three adults in UK with pathologically-confirmed HIV-associated CD8 encephalitis (HIV-CD8E).

**Case**	**Age/sex**	**Previous (months)**		**Current**			**ART**	**CPE score**	**Risk event category**	**Clinical presentation**	**Imaging**	**CSF**			**Cortico-steroids**	**Outcome**	**Diagnosis mode**
		**CD4 c/μL**	**pVL c/mL**	**CD4 c/μL**	**CD8 c/μL**	**pVL c/mL**						**Cells**	**Protein g/L**	**VL c/mL**			
1^a^	32/F	122 (3)	1,193 (3)	95	ND	291	Stopped Z, Em, s/L 3 m previously	8	4	17 d headache, confusion	Typical	12 ly	0.4	ND	No	Died of HIV-CD8E	Autopsy
2	40/M	547 (4)	19,126	543	NA	21,359	Nil	0	6	28 d headache, confusion,	Typical	17 ly	1.35	ND	Yes	Died of HIV-CD8E	Autopsy
3	28/F	847 (3)	<50 (3)	876	2,001	<50	D, Z, r/L	9	1	7 d headache, confusion	Typical	20 ly	ND	ND	No	Died of HIV-CD8E	Autopsy
4	46/F	360 (4)	2,600 (4)	170	960	86,800	stopped A, T, r/S 4 m previously	5	3	Cardiac arrest	ND	ND	ND	ND	No	Died of HIV-CD8E	Autopsy
5	40/M	NA	NA	315	306	<50	F, r/S	3	2	10 d confusion	Typical	ND	ND	ND	No	Died of HIV-CD8E	Autopsy
6	36/M	847 (3)	<50 (3)	>400	NA	3,568	D, Z, r/L	9	3	7 d headache, confusion, fits	Typical	ND	ND	ND	No	Died of HIV-CD8E	Autopsy
7	43/F	410 (4)	<50 (4)	240	1,290	<50	T, D, r/A	5	1	21 d headache, TIA	Typical	8 ly	0.68	ND	No	Died of HIV-CD8E	Autopsy
8	47/F	1,030 (5)	<50 (5)	824	1,755	238	r/A	2	3	7 d headache, confusion	Typical	NA	>0.8	ND	No	Died of HIV-CD8E	Autopsy
9^b^	49/F	521 (4)	28,808 (4)	374	970	12,062	T, A, r/A	6	4	28 d cognitive impairment	Typical	ND	ND	ND	No	Survived	Brain biopsy
10	29/F	530 (3)	NA	560	960	<50	L, A, r/L	7	1	5 d headache, obtunded	Typical	ND	ND	ND	No	Died of HIV-CD8E	Autopsy
11	41/F	NA	151,544 (3)	266	NA	439	A, M, R, r/L	12	3	4 d headache, cardiac arrest	ND	ND	ND	ND	No	Died of PE following recovery	Autopsy
12	44/M	298 (3)	<50 (3)	233	896	<50	E, r/D	5	1	28 d headache, 17 d confused	Typical	80 ly	1.2	<50	Yes	Died of HIV-CD8E	Autopsy
13	37/F	1,032 (5)	<50 (5)	353	880	8,759	stopped T, Em, r/S 1 m previously	5	2 + 3	28 d headache, 6 d vomiting	Typical	ND	ND	ND	No	Died of HIV-CD8E	Autopsy
14	19/M	10 (4)	NA	64	NA	600	T, Em, Ef	7	4	21 d limb paraesthesia 7 d seizures	Typical	ND	ND	ND	Yes	Survived	Brain biopsy
15	33/F	370 (3)	340 (7)	200	1,340	8,300	Nil	0	6	Found dead (at home)	ND	ND	ND	ND	No	Died of HIV-CD8E and DILS	Autopsy
16	51/F	1,024 (5)	<50 (5)	ND	ND	ND	T, Em, r/D	7	2	4 d headache, vomiting	Typical	ND	ND	ND	No	Died of HIV-CD8E	Autopsy
17^c^z	52/F	870 (3)	<50 (3)	220	360	<50	T, Em, r/A	6	2	21 d headache, confusion, drowsy	Typical	ND	ND	1,100 = VE	Yes	Survived	Brain biopsy
18	33/F	450 (11)	<50 (11)	348	741	3,300	M, r/D	9	3	28 d headache, cardiac arrest; second trimester pregnancy	Typical	NA	NA	7,700 = VE	No	Died of HIV-CD8E	Autopsy
19	35/F	594 (3)	91,201 (3)	487	703	125,893	stopped r/D 5 m previously	3	3	14 d headache, confusion	Typical	ND	ND	ND	Yes	Survived	Brain biopsy
20	52/F	1,283 (8)	460 (2)	ND	ND	ND	T, Em, c/D	7	1	10 d headache, vomiting, ataxia, coned	Typical	ND	ND	ND	No	Died of HIV-CD8E	Autopsy
21^a^	45/M	50 (2.5)	838,000 (2.5)	320	ND	4,000	E, L, Z	8	4V	7 d worsening of long-standing cognitive decline: 2 d generalized seizures	Typical	23 ly	1.15	ND	No	Died of HIVE and HIV-CD8E	Autopsy
22	45/M	520 (12)	<50 (7)	ND	ND	ND	L, M, r/A	7	2	3 d worsening of long-standing cognitive decline	ND	ND	ND	ND	No	Died of HIV-CD8E	Autopsy
23	69/M	400 (13)	<50 (13)	460	NA	<50	T, Em, N	8	1V	10 d headache, confusion 2 d seizures	Typical	26	0.56	<50	Yes	Died of HIV-CD8E	Autopsy

Previously published references:

a *(1)*;

b *(13)*;

c *(11). Key: d, days; m, months; c/μL, cells/μL; pVL, plasma viral load (c/mL: copies/mL); VL, viral load (c/mL: copies/mL); ND, not done; NA, not available; ART, antiretroviral therapy; A, abacavir; c/D, cobicistat-boosted darunavir; D, didanosine; E, etravirine; Ef, efavirenz; Em, emtricitabine; F, fosamprenavir; L, lamivudine; M, maraviroc; N, nevirapine; r/A, ritonavir-boosted atazanavir; r/D, ritonavir-boosted darunavir; r/L, ritonavir-boosted lopinavir; r/S, ritonavir-boosted saquinavir; R, raltegravir; T, tenofovir; Z, zidovudine; PE, pulmonary thromboembolism; TIA, transient ischaemic attack; HIV-CD8E, HIV-CD8 encephalitis; VE, CSF viral escape; ly, lymphocytes; HIVE, HIV encephalitis; DILS, diffuse infiltrative lymphocytosis syndrome. Risk event: 1, well-controlled HIV infection, occurring without identified risk event; 2, intercurrent infection or malignancy; 3, ART treatment interruption/poor adherence; 4, IRIS after commencing ART; 5, ART drug resistance; 6, not in receipt of ART; V, variant*.

Predominant clinical features included headache in 17 patients (duration between 4 and 28 days), and confusion in 10 patients (duration 7–28 days) and progressive cognitive decline in 3 patients (Table 1). All but one of those who lapsed into unconsciousness died during that episode: one patient (#11) recovered from HIV-CD8E coma (without corticosteroid therapy), but died later from venous thromboembolism. One patient was pregnant (2nd trimester at time of death), and one (who had not received ART) was found dead at home. Sixteen of 17 (94%) patients who did not receive corticosteroids died, as did three of six patients who received corticosteroids: *p* = 0.04 (Two-tailed Fisher exact test).

#### Imaging

**Figure 1:**
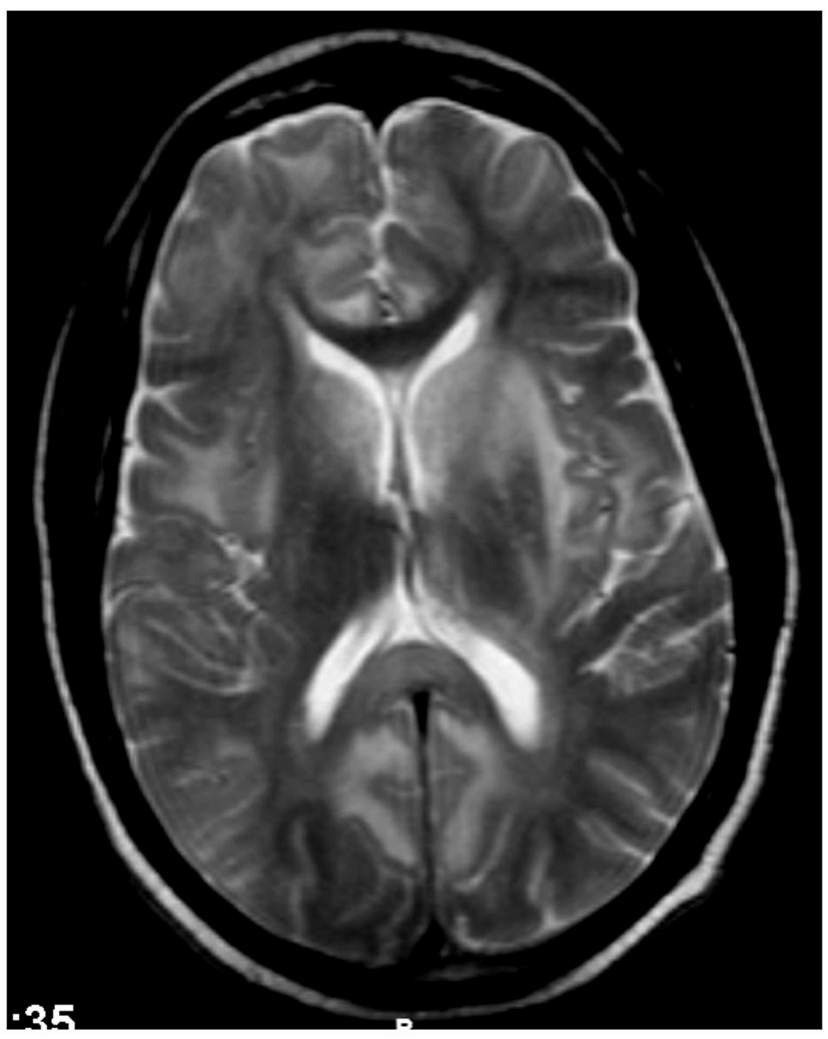
Case 12: Brain MR imaging (axial T2-weighted image) demonstrating multiple confluent white matter hyperintensities. There are significant hyperintensities bilaterally in the caudate, partly extending into the adjacent anterior putamen but sparing the thalami. The genu of corpus callosum is strikingly spared but the splenium/posterior body shows hyperintensities.

**Figure 2:**
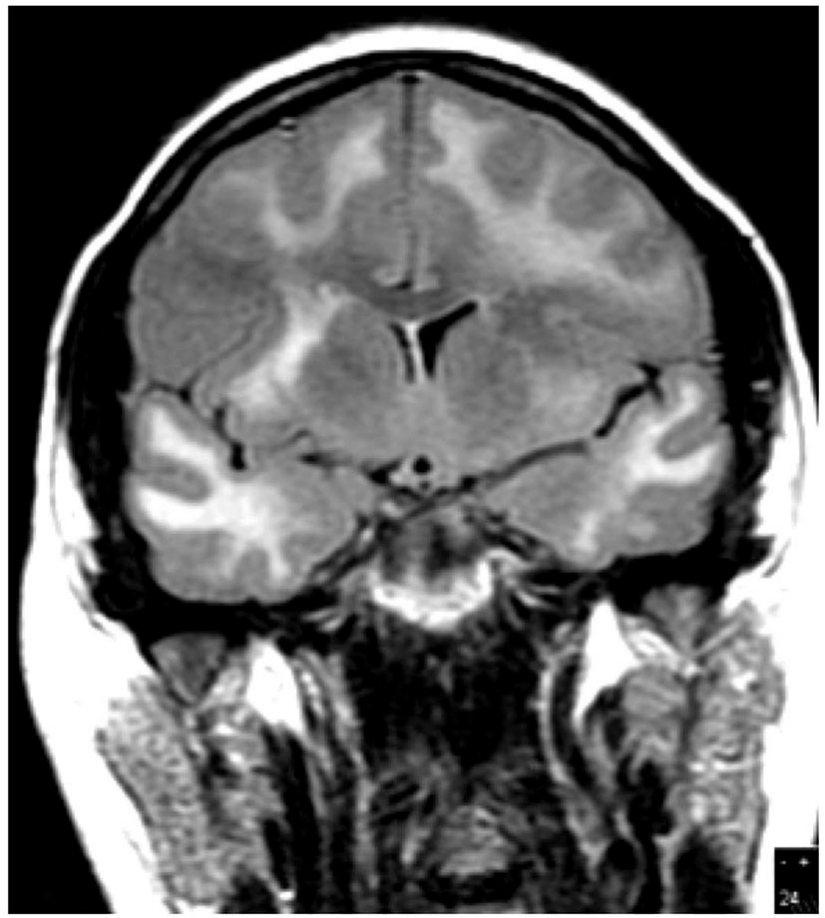
Case 7: Brain MR imaging (coronal T2-weighted image) demonstrating extensive white matter hyperintensity.

In 19 patients, cranial MR imaging showed multiple, bilateral, confluent, and symmetrical high signal intensities, localized throughout the cerebral and cerebellar white matter, and pons, with or without involvement of deep gray matter on both T2 and fluid-attenuation inversion recovery (FLAIR) sequences (Figures 1, 2). When done, diffusion-weighted imaging (DWI), showed marked diffusion restriction, particularly evident at the periphery of the confluent white matter signal abnormalities. In some patients, post-gadolinium T1 sequences showed multiple gadolinium-enhancing lesions as previously described (1, 7, 11). These MR imaging appearances are distinguishable from those seen in (1) HIVE: symmetrical periventricular and deep white matter hyperintensities on T2-weighted images, relative sparing of the sub-cortical white matter, without mass effect or enhancement; (2) progressive multifocal leukoencephalopathy: asymmetrical, multifocal periventricular and sub-cortical (U-fibers frequently involved) white matter, hypointense on T1 and hyperintense on T2, without mass effect; and (3) primary CNS lymphoma: typically seen as a T1-hypointense gadolinium-enhancing lesion, frequently showing sub-ependymal involvement.

#### Laboratory

**Figure 3:**
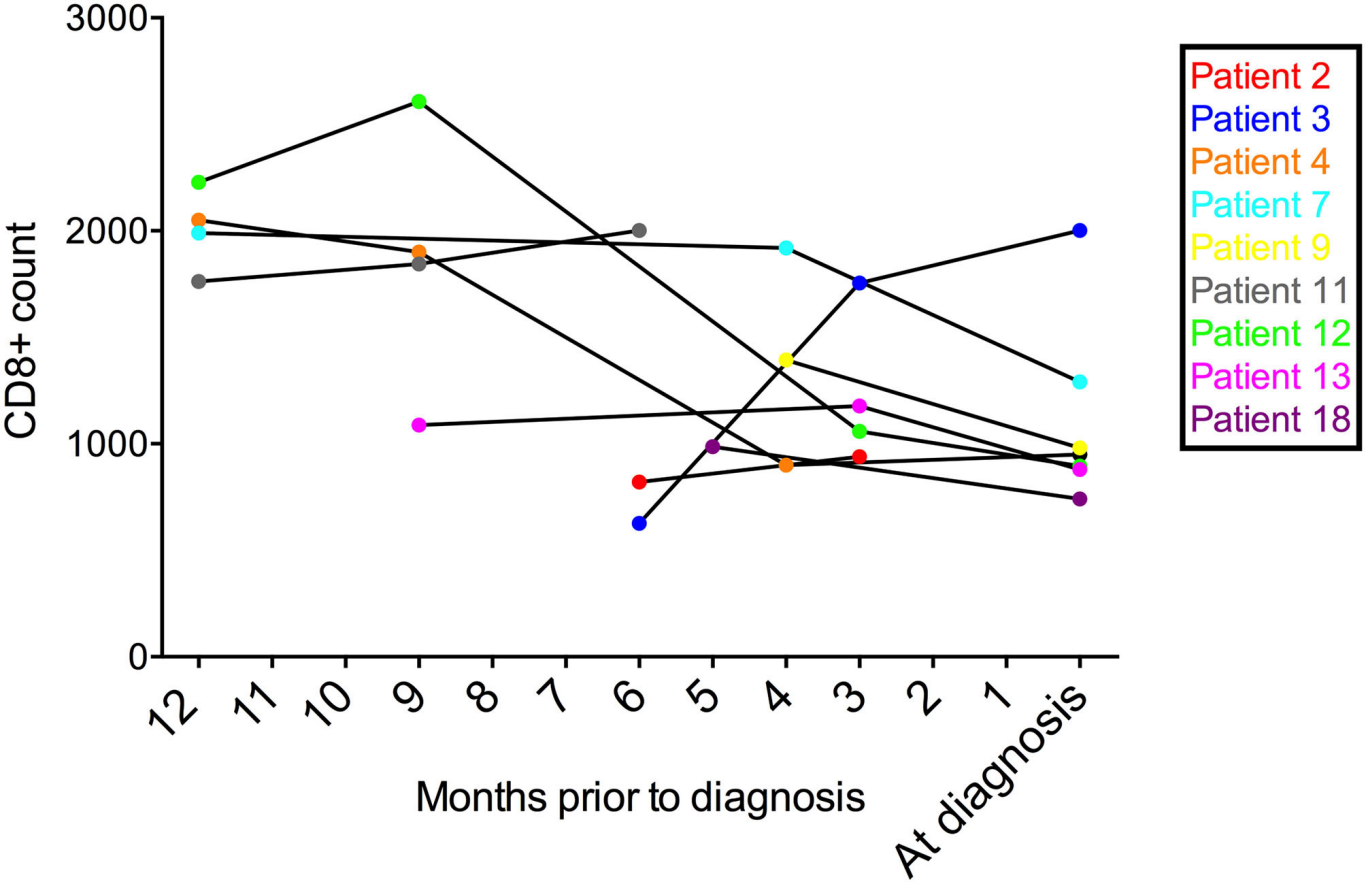
Graph of blood CD8^+^ T-cell counts from nine patients over the year prior to presenting with HIV-CD8E.

The median CD4+ T-lymphocyte count (available in 20 patients), obtained within the year before presentation with HIV-CD8E, was 523 cells/mm^3^ (range 10–1,283), and at presentation with HIV-CD8E (19 patients) was 327 cells/mm^3^ (range 64–876) (Table 1). CD8^+^ T-lymphocyte counts were available in 13 patients at diagnosis of HIV-CD8E and ranged between 306 and 2001 (median = 960 cells/mm^3^: normal range 250–999). Of the 3 patients with a blood CD8^+^ T-lymphocyte count >1,000 cells/mm^3^, one (#15) had diffuse infiltrative lymphocytosis syndrome (DILS) (14) at autopsy. Nine patients had more than one peripheral blood CD8^+^ T-cell count available in the months prior to presentation with HIV-CD8E: in all but one, these remained stable or dropped prior to death (Figure 3).

Ten patients underwent CSF examination. CSF samples for lymphocytes (not sub-typed) were available in seven patients (Table 1). Median CSF lymphocyte count was 20 (range 8–80). Of the 3 patients with data available, 2 (#17 and 18) had a higher viral load in CSF than in contemporaneous plasma, i.e., they had viral escape (6); two (#12 and 23) did not. In the one patient tested (#17) there was no evidence of HIV drug resistance in CSF. The single patient tested did not have autoimmune encephalitis-associated antibodies.

#### Histopathology

**Figure 4:**
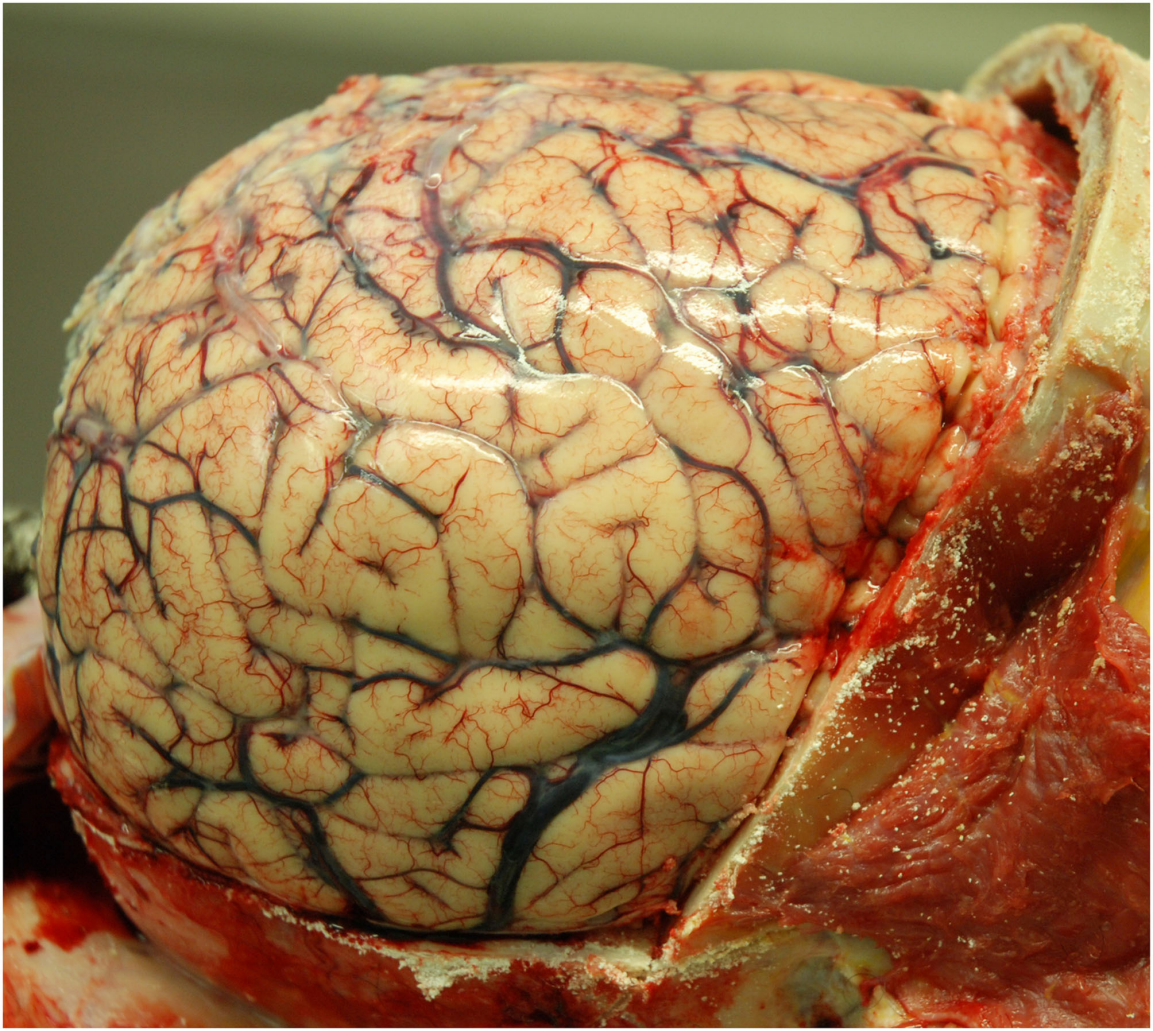
Case 10. Uniformly swollen brain in a patient with HIV-CD8E (gyral flattening and swelling) after removal of the skull bone [(2), reproduced with permission].

**Figure 5:**
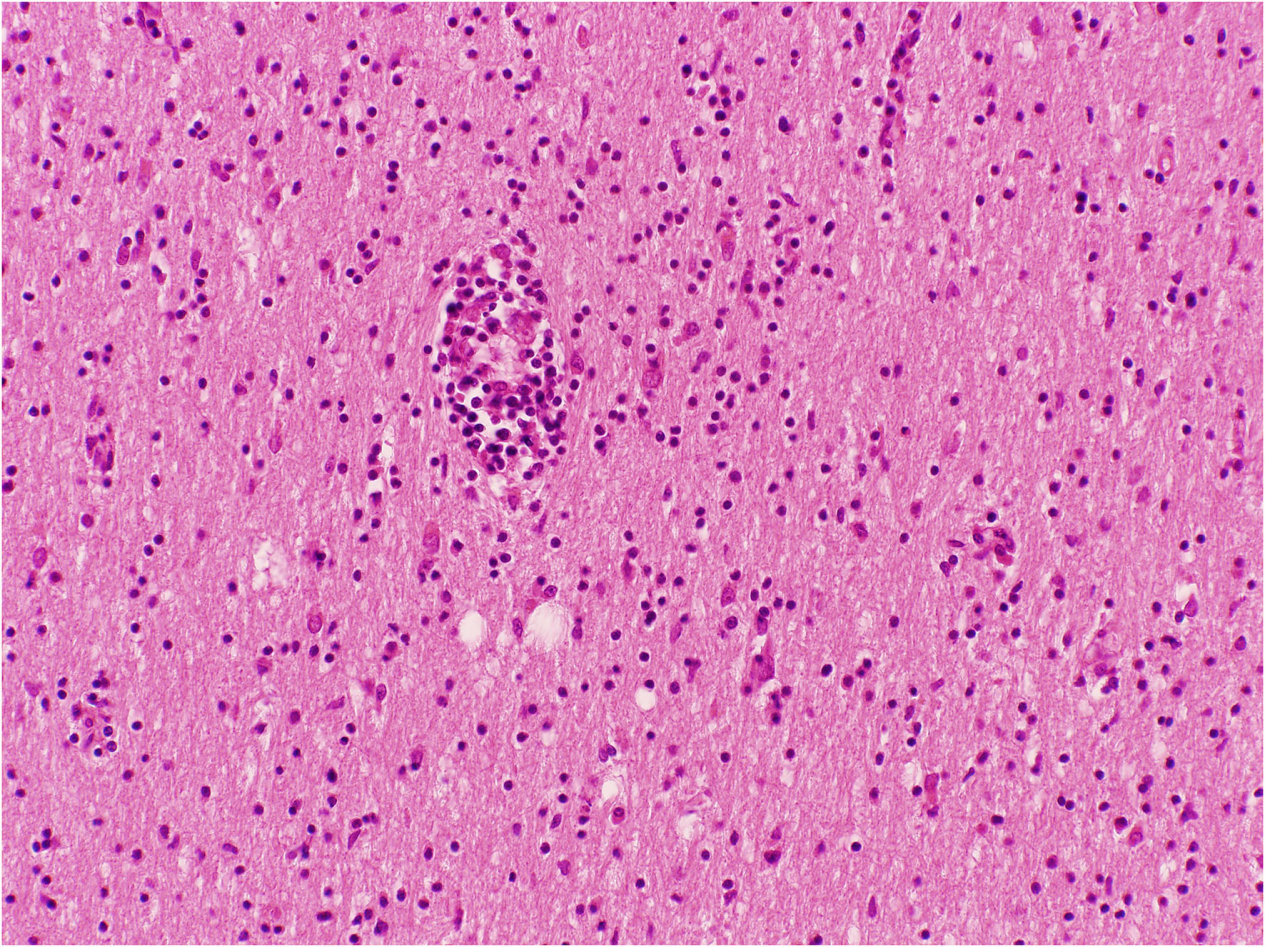
Cerebral white matter with perivascular and diffuse lymphocytes in the neuropil; Haematoxylin and Eosin stain. Original magnification × 100.

**Figure 6:**
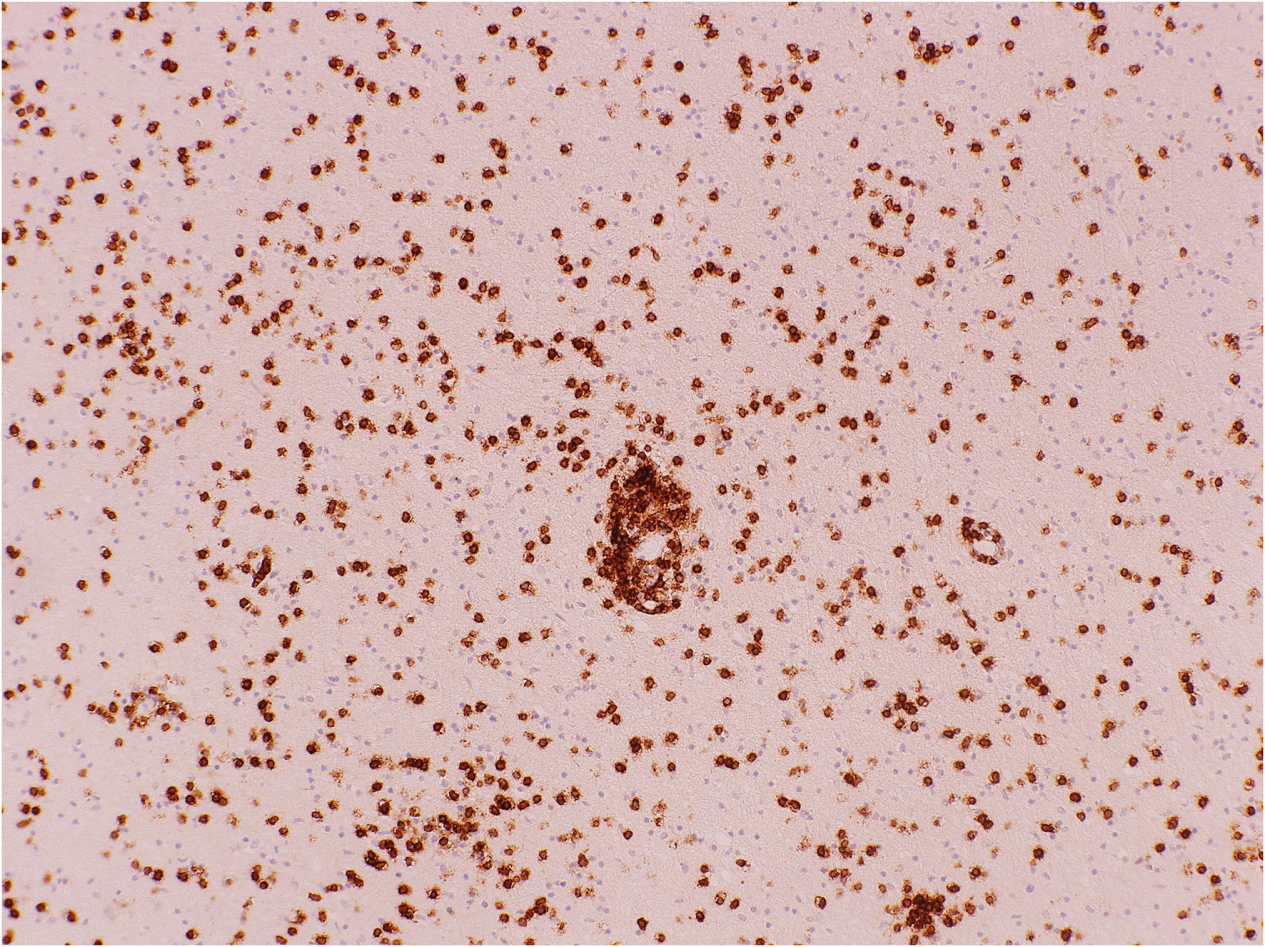
Cerebral white matter: CD8^+^ T-cells demonstrated with immunohistochemistry (antibody against CD8). Original magnification × 100.

**Figure 7:**
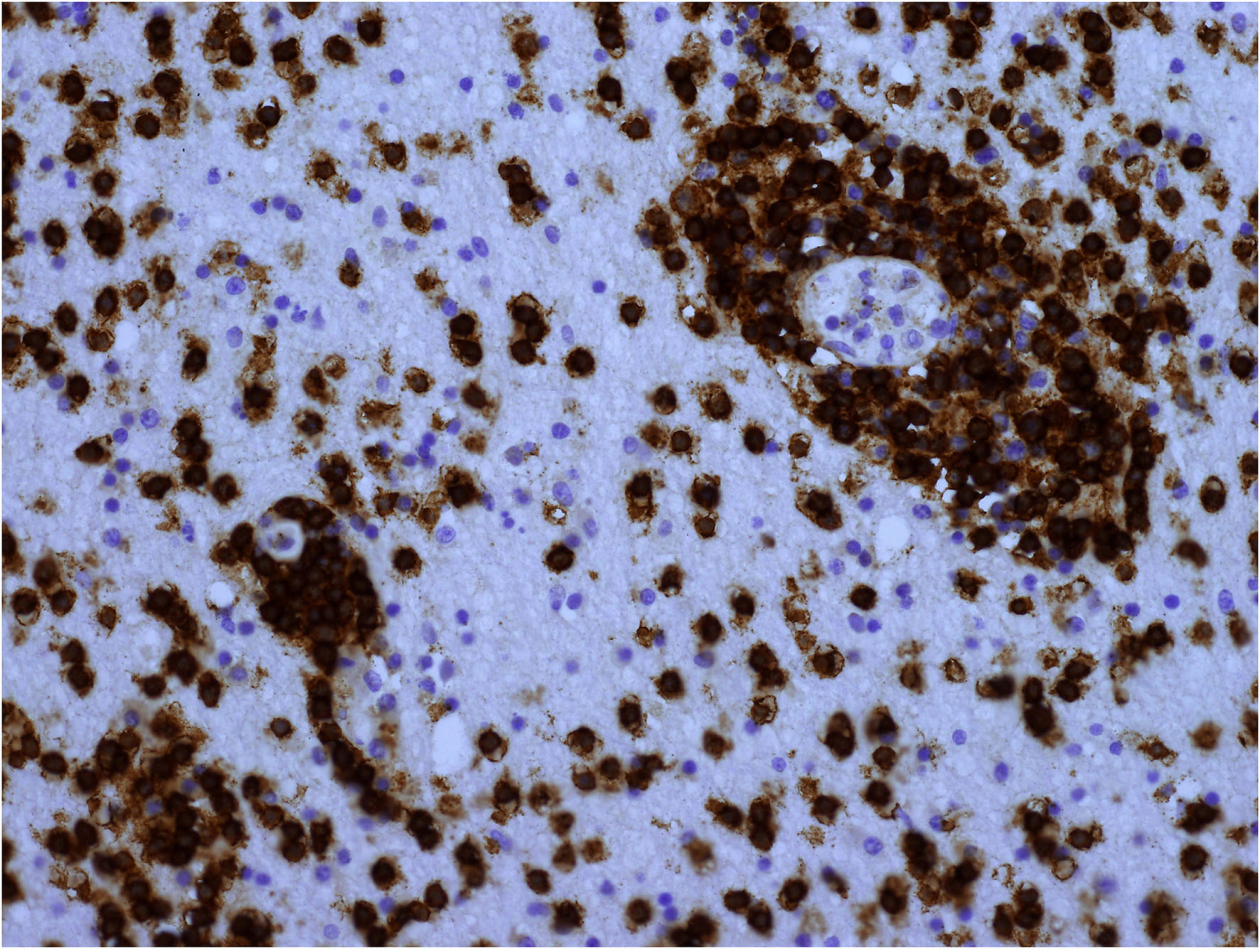
Cerebral white matter CD8^+^ T-cells: the highest density seen in a UK patient. Original magnification × 400.

**Figure 8:**
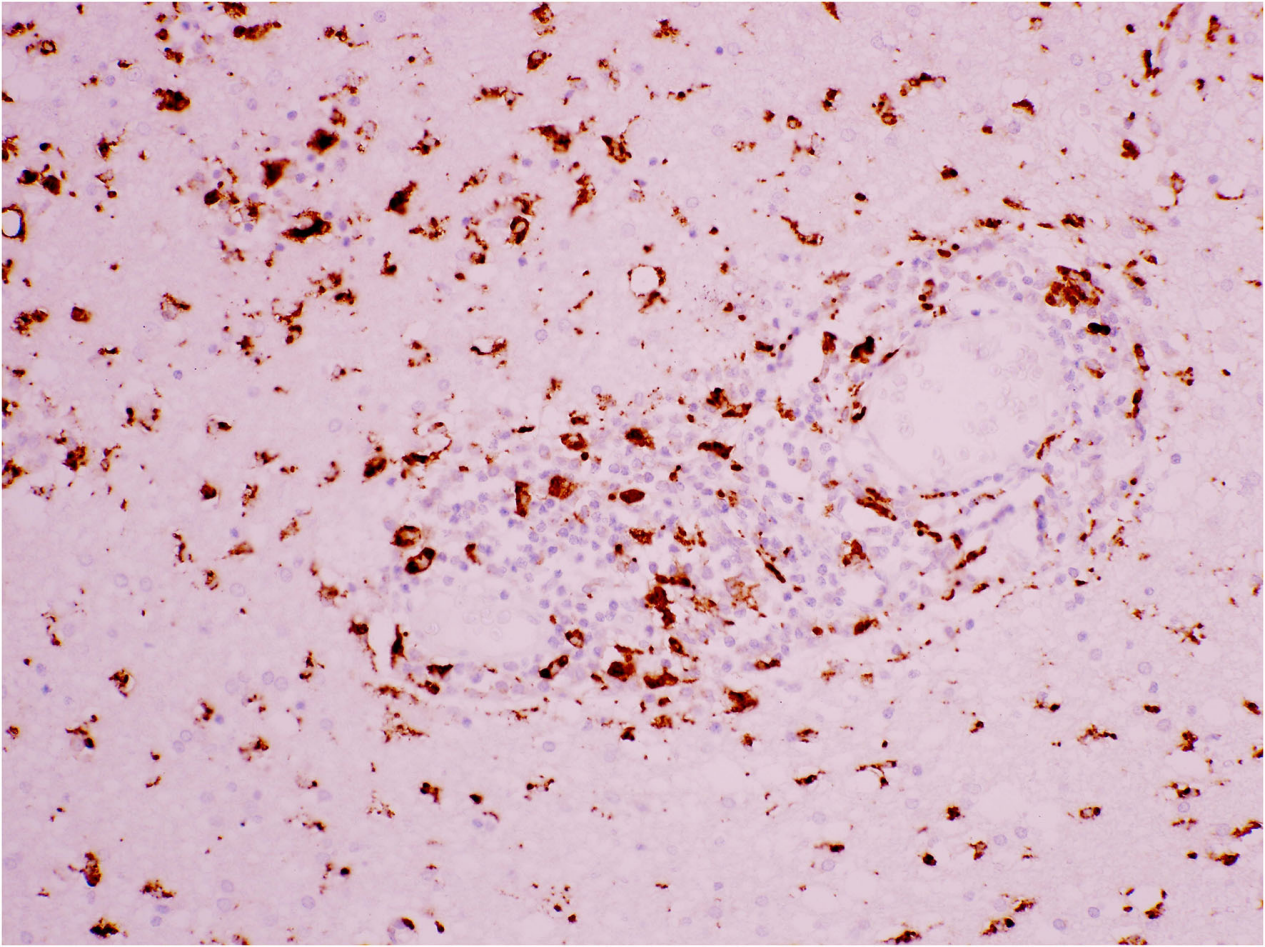
Cerebral white matter: activated CD68^+^ microglial cells around a vessel and through the neuropil, demonstrated with immunohistochemistry (antibody against CD68). Original magnification × 200.

**Figure 9:**
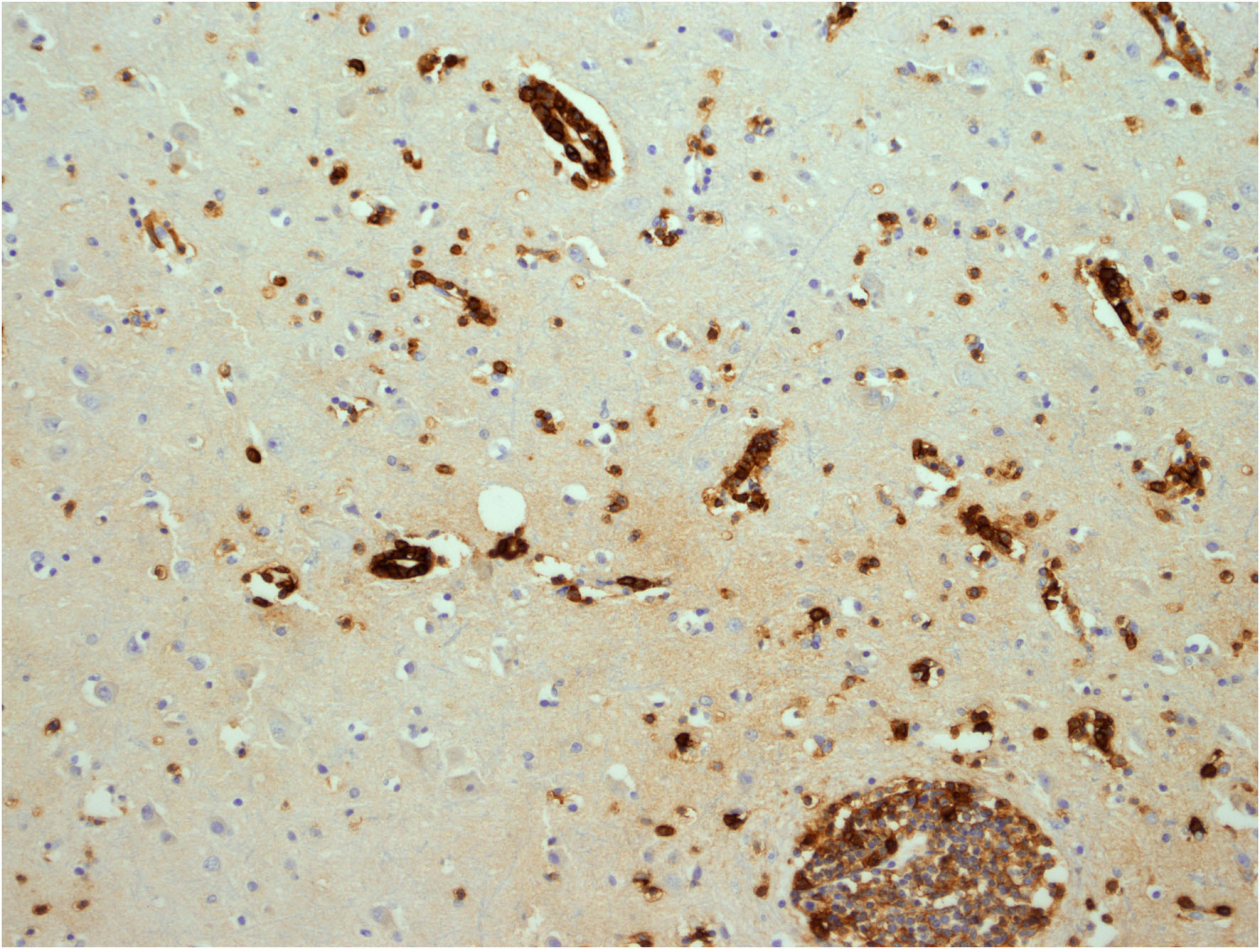
Cerebral white matter: CD38^+^ activated T-cells, demonstrated with immunohistochemistry (antibody against CD38). Original magnification × 200.

**Figure 10:**
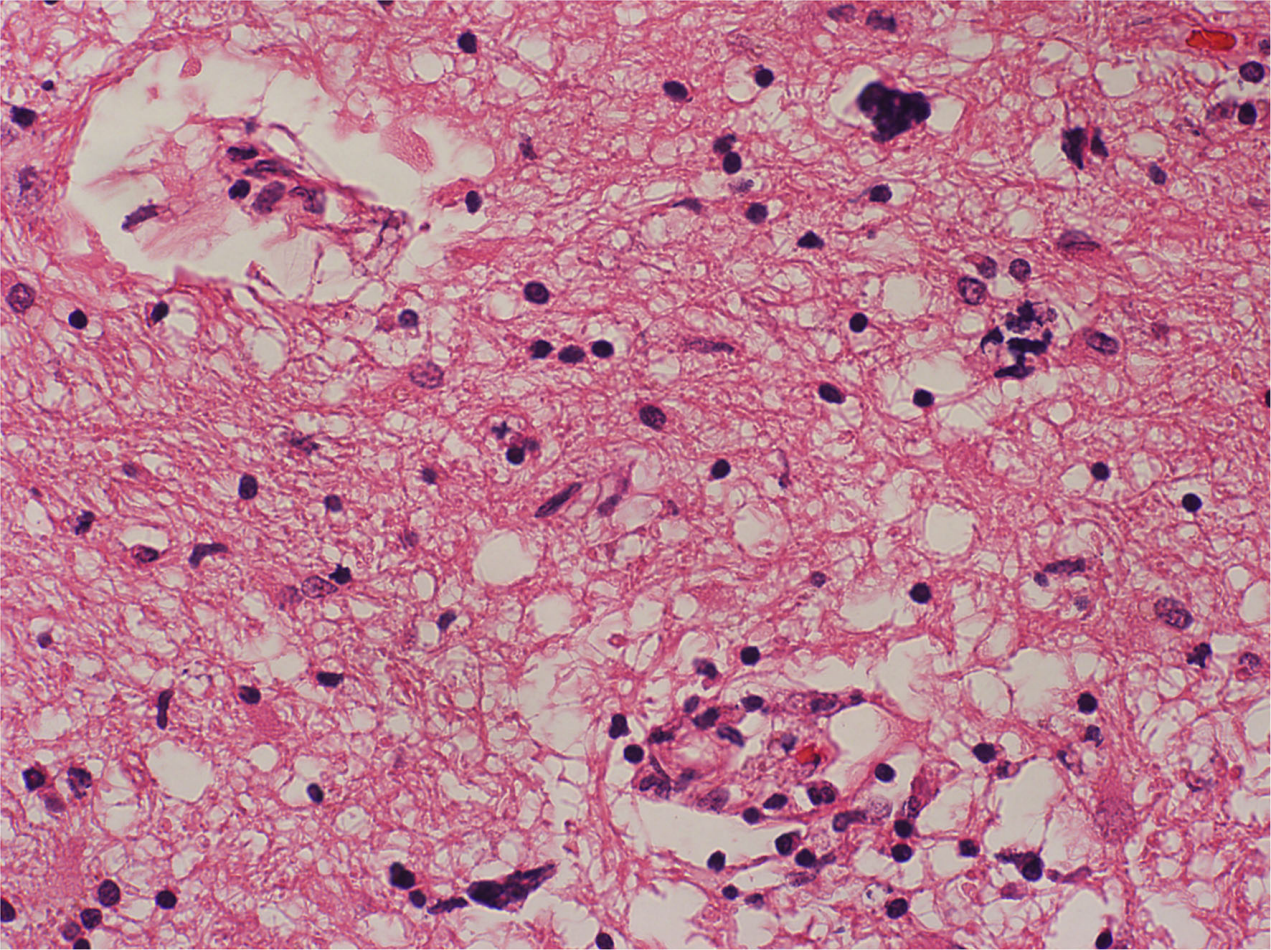
Cerebral white matter with three shrunken multinucleate giant cells; this is inactive variant HIV-CD8E (Case 23). Also present are lymphocytes, enlarged astrocytes, and microglia. Haematoxylin and Eosin stain. Original magnification × 200.

Histopathological examination was performed on 19 autopsy brains, and 4 brain biopsies. The autopsy brains were diffusely swollen (Figure 4). The diagnosis of HIV-CD8E was based on brain histopathology: perivascular and diffuse cerebral infiltration by CD8^+^ T-cells—in the white matter always and less intense in gray matter cortex, involving the cerebrum, brain stem and cerebellum and, where available, the upper segment of the spinal cord white matter. Moderate lymphocytic meningitis was present in all cases. Microglial activation (identified from H&E stain and CD68 IHC) was always present, but astrocyte activation, as judged by GFAP IHC, was mild. Other specific infectious causes of encephalitis were excluded (Figures 5–10). We did not have sufficiently detailed data available to correlate pre-mortem imaging with any specific brain areas' pathology; the histopathology of all brain areas was similar independent of the risk factors for HIV-CD8E, although in some patients (e.g., #23, with temporal lobe disease predominant) some cortical areas appeared more affected than others.

Diagnostic exclusion criteria for HIV-CD8E included: ischaemic stroke, vasculitis, HIVE (multinucleate giant cells and identifiable HIV antigens on IHC) (4) without associated CD8E, encephalitis with viral inclusions (including HSV, CMV, and JC virus), bacterial, fungal, and tuberculous meningo-encephalitis, toxoplasmosis, and lymphoma. In HIV-CD8E, perivascular T-cell infiltration is present and T-cells are also seen passing through the vessel walls, but true vasculitis, such as HIV-vasculitis with stroke, with or without necrosis and granulomas, is not a feature of HIV-CD8E.

In the 10 autopsy cases, where they were done, the CD8^+^ T-cells were CD38^+^ (i.e., activated) but CD56 immunostain for natural killer cells was negative. CD4^+^ T-cells were present in small numbers, and CD20^+^ B-cells were prominent in only one case (#9). Only one of the brains (#21) had identifiable HIV-1 virus using HIV p24 IHC, in conjunction with microglial giant cell nodules and diffuse CD8^+^ T-cell infiltrate (termed “variant HIV-CD8E” with HIVE). Another brain (#23) had microglial giant cell nodules without HIV p24 positive IHC). All the others had neither demonstrable HIVp24, nor microglial nodules, nor microglial giant cell encephalitis.

One autopsied patient (#15) had DILS (14), with diffuse CD8^+^ T-cell infiltration of the spleen, lungs, liver and kidney, in addition to severe HIV-CD8E.

Of the five patients with intercurrent infection, one had pulmonary tuberculosis (#5), two (#13 and #16) had community-acquired pneumonia, patient #17 had leg cellulitis, and #22 had urinary tract infection confirmed microbiologically.

#### Non-histopathology Microbiological Investigations

Where tested, CSF and brain tissue viral studies were negative for all tested viruses (adenovirus, enterovirus, HSV, HHV6, HHV8, VZV, EBV, CMV, JC, West Nile, parvovirus) except HIV-1. Four cases had T-cell clonality studies performed on formalin-fixed paraffin-embedded brain tissue by detection of gene rearrangements: none showed a single dominant clone. Mixed bi-clonal and oligoclonal peaks within polyclonal backgrounds were observed. No T-cell lymphomas were identified. Patients #1 and #21 had positive PCR analysis for HIV on autopsy brain tissue (1).

#### Risk Categories for HIV-CD8E in the 23 Patients

The risk categories for development of HIV-CD8E are shown in Table 1. In summary, in six patients ART had recently been interrupted (one also had an intercurrent infection), in six there was no evident risk event, the patients having well-controlled HIV infection on ART, in five others there was an intercurrent infection, three had HIV-CD8E as an IRIS, presenting after initiation of ART, two patients had never received ART, and one, interpreted as IRIS, had active “variant” HIV-CD8E.

### Global Published Cases

We found 34 cases of HIV-CD8E with histological diagnosis reported in the literature from 2002 to 2021 (Table 2). The pathological descriptions in the literature parallel those found in the UK cases, with global brain CD8^+^ T-cell infiltration. Some reports mention describe some CNS areas being more affected, e.g., spinal cord (22) and occipital lobe (25).

**Table 2:** Previously published non-UK (global) histopathologically-confirmed cases of HIV-associated CD8 encephalitis.

**Year of report**	**Country of report**	**Patient ethnicity**	**Histopathology**	**Outcome**	**Risk category**	**CSF viral escape**	**Corticosteroids given**	**References**
2006	USA	NA	Biopsy	Alive	3	Yes	Yes	(15)
2008	USA	Hispanic	Autopsy	Died	4	ND	No	(16)
		African	Biopsy	Survived	4	ND	No	
2009	Germany	African	Autopsy	Died	4	ND	Yes	(17)
2009	Canada	Africa	Biopsy	Alive	4	ND	Yes	(18)
2010	Germany	African	Autopsy	Died	4	ND	Yes	(19)
2011	USA	NA	Biopsy	Alive	4	ND	Yes	(12)
2012	USA	NA	Biopsy	Alive	1	Yes	No	(5)
		NA	Biopsy	Alive	5	Yes	No	
2013	France	African	Biopsy	Alive	3 + DILS	ND	Yes	(20)
2013	France	Of 14 patients (only 10 had histo-pathology): 7 were sub-Saharan African, 2 were North African, 5 were Caucasian; but individual ethnicity data are not presented.	Biopsy and Autopsy	Died	2	ND	Yes	(7) (cases #1–10)
			Biopsy	Alive	3	ND	Yes	
			Biopsy	Alive	4	No	Yes	
			Biopsy	Died	2	Yes	Yes	
			Biopsy	Died	4	ND	Yes	
			Biopsy	Died	3	Yes	Yes	
			Biopsy	Alive	2	ND	Yes	
			Biopsy	Alive	6 and 2	ND	Yes	
			Biopsy	Alive	3	No	Yes	
			Biopsy	Alive	1	Yes	Yes	
2013	France	NA	Biopsy	Alive	4	ND	Yes	(21)
2014	France	African	Biopsy	Alive	3	No	Yes	(22)
2016	Japan	Japanese	Biopsy	Alive	5	Yes	No	(8)
2018	USA	NA	Biopsy	Alive	1	Yes	No	(23)
2019	USA	African	Biopsy	Alive	6	ND	Yes	(9)
		Hispanic	Biopsy	Alive	3	ND	Yes	
2019	India	Indian	Biopsy	Died	3	ND	Yes	(24)
2020	Japan	Japanese	Biopsy	Alive	4	ND	Yes	(25)
2020	Ireland	African	Biopsy	Alive	5	Yes	Yes	(26)
		African	Biopsy	Alive	7	ND	Yes	

*Key: NA, not available*.

*Risk category: 1, well-controlled HIV infection, HIV-CD8E occurring without identifiable risk event; 2, intercurrent infection or malignancy; 3, ART treatment interruption/poor adherence; 4, IRIS after commencing ART; 5, ART drug resistance; 6, not in receipt of cART; 7, EBV-associated*.

*The Critical review of the stated risk events resulted in changes in some instances from the original categories*.

*The patients in Lescure et al. (7) and Gray et al. (21) series overlap*.

Four of these were UK cases included in our series above, and hence are excluded from this section to avoid duplication. A further 15 cases have been reported on the basis of clinical signs and symptoms, consistent imaging, and exclusion of alternative diagnoses; but lacking definitive neuropathology. These are not congruent with the proven cases and are not considered further—see Supplementary Table 1.

Table 3 summarizes all 53 cases of HIV-CD8E (i.e., the UK series and global published cases combined) in terms of risk categories and whether CSF viral escape was demonstrated. Treatment interruption (*n* = 14) and IRIS (*n* = 14) were the commonest risk events, followed by those without an identified risk event (*n* = 9), and intercurrent infection (*n* = 7). Three patients had ART drug resistance, and four had not been treated with ART. Two patients had more than one risk event.

**Table 3:** Patients with pathologically proven HIV-CD8 encephalitis: risk event categories and CSF viral escape; summary data from UK 23 cases and from 30 global previously published cases.

**Risk event category**	**CSF viral escape: data not available**	**CSF viral escape**	**No CSF viral escape**	**Total**
1. No event identified	4	3	2	9
2. Intercurrent infection	5	2	0	7
3. Treatment interruption/ poor adherence	9	3	2	14
4. IRIS	13	0	1	14
5. ART drug resistance	0	3	0	3
6. Not in receipt of ART	3	0	0	3
7. EBV-associated	1	0	0	1
6 + 2	1	0	0	1
2 + 3	1	0	0	1
Total	37	11	5	53

CSF examination was reported in only 16/53 (30%) of HIV-CD8E cases, as cerebral swelling precluded CSF examination in the majority. Of those tested, 11 (68%) had demonstrated viral escape, and five (31%) did not (Table 3).

Overall, 25/53 (47%) patients died. Nine of 30 (30%) patients who received steroids died, as did 16 of 23 (70%) that did not receive steroids: *p* = 0.005 (Two tailed Fisher exact test).

#### Ethnicity of HIV-CD8E Patients

Twenty-one of the 23 patients in the UK series were of Black African ancestry. The global literature also indicates a majority (58–75%) with Black African ethnicity where data are stated (Table 2). The exact proportion cannot be calculated as one series (7) states ethnicity only for the overall set (*n* = 14), and only 10 patients had histopathological confirmation of HIV-CD8E.

## Discussion

HIV-associated CD8 encephalitis (HIV-CD8E) remains a poorly understood condition with relatively few reported cases in the literature. The first reported patient with fatal HIV-CD8E died in 2002 (1)—Case #1 in the present series. The earliest documented fatalities were Case #1 in Lescure's et al. series (7) and Case #3 in the Kerr et al. series (26) both dying in 2001. The UK case series augments reported cases with histopathological confirmation globally to 53. Combining our series with other published cases to date revealed a number of important risk factors which have not been previously emphasized, including the fact that most cases of HIV-CD8E occur in people of Black ethnicity, and the frequent associated finding of CSF viral escape. Other risk factors are treatment interruption, intercurrent infection, IRIS, drug resistance and, possibly, EBV infection in the brain (26). There are patients whose HIV is well-controlled and do not have any evident risk factors; patients not on ART who develop HIV-CD8E; and rare patients with co-occurrence of HIV-CD8E with HIV-encephalitis (HIVE) which we term “variant-HIV-CD8E.” We do not consider CSF viral escape to be an independent risk factor *per se*, since it is found across the spectrum of precipitating factors with the exception of IRIS; rather, this may be a linked phenomenon driving the pathological process as discussed below.

A satisfactory pathogenesis to explain the clinico-pathological phenomenon of HIV-CD8E needs to accommodate: 1. The predominant Black African ethnicity of the patients; 2. The curious epidemiology of HIV-CD8E—a mainly European phenomenon, seemingly missing in Africa and relatively under-reported in USA; 3. The widely differing immune status and HIV viral loads in patients at presentation, including those well-controlled, those with rising plasma viral load (pVL) (as in drug resistance, intercurrent infection, treatment interruption), those with falling pVL (as in IRIS), those never on ART, and those patients both with and without demonstrable CSF viral escape; 4. The clear association between use of corticosteroid therapy in life and survival.

A pathogenesis also needs to consider the pathological contribution and impact of the known risk factors, and also whether HIV-CD8E is part of a spectrum of other defined HIV-related CNS disorders such as HIV-associated neurocognitive disorders (27).

### Ethnicity

The preponderance of patients with Black African ancestry and HIV-CD8E suggests a genetic linkage to development of HIV-CD8E, involving genetically-determined cell-mediated immune response to viral antigens, or possibly differences in blood-brain barrier function. For comparison, there is the known linkage of the risk of collapsing glomerulopathy including HIV-associated nephropathy (HIVAN) in African patients who have G1 and G2 polymorphisms of the APOL1 gene, which are protective against trypanosomal disease (28). Another possibility is that the preponderance of Black ethnicity in European settings relates to differences in ART adherence, HIV factors such as nadir CD4, and comorbidities in this population. Our data do not permit determination of whether host genetics, HIV factors, or both, are responsible.

### Epidemiology

Globally, the largest number of individuals with HIV infection reside in sub-Saharan Africa. Recent reviews of HIV-related brain disease in Africa do not describe cases of HIV-CD8E (29, 30), however HIV-CD8E may be under-recognized and under-reported in this setting. Between 2018 and 2020, SN has observed several clinico-radiologically-diagnosed cases of HIV-CD8E among Black African ART-treated patients in Cape Town, South Africa (personal observations, unpublished). A large systematic HIV autopsy survey in adult Africans with HIV-1, HIV-2 and dual-infections from Cote d'Ivoire in the pre-ART era included brain pathology (31). That study showed that typical HIVE was very uncommon and no one had HIV-CD8E.

One explanation may be the relative rarity of the syndrome. In England and Wales, where there is an overall autopsy rate approaching 20% of all deaths (Coroner Statistics, 2000–2019), only one fatal case per year has been pathologically diagnosed in the context of >500 deaths among adults with HIV infection each year. By contrast, in Africa, investigation of medical deaths with autopsy (but not the less stringent forensic autopsies) is uncommon. Thus, it is likely that such deaths are occurring but are not autopsied or recognized.

### Treatment Status, Viral Load, and CSF Viral Escape

In the combined series of 53 patients, common risk events were treatment interruption (*n* = 14) and IRIS (*n* = 14), followed by those without an identifiable risk (*n* = 9) and intercurrent infection (*n* = 7). Most patients did not have CSF examined, likely due to cerebral swelling making lumbar puncture potentially unsafe. Of the 16/53 (30%) that had CSF results available, 11 (68%) had demonstrated viral escape. Among five well-controlled patients, three had viral escape and two did not. CSF resistance profiles were not reported, but two patients had ART drug resistance detected peripherally; both had demonstrated CSF viral escape. Three out of five with treatment interruption and with CSF viral loads measured had viral escape, as did two patients with intercurrent infection. Of those with IRIS as their risk factor, the single patient tested did not have CSF viral escape.

CSF viral escape is a marker of disparity between HIV viral load in blood and brain (32). The association between HIV-CD8E and CSF viral escape has not been previously highlighted; for example a previous series of 14 patients with HIV-CD8E (7) reported that only two (14%) were associated with CSF viral escape, although re-inspection of their data reveals that viral escape was present in 3 further patients. However, there is no consistent pattern of viral escape across all of the risk events for HIV-CD8E.

### “Variant” HIV-CD8E

Case #21 in the UK series reflects some features of a previous report. Langford et al. (33) described five patients with chronic HIV-associated dementia who died of pneumonia or sepsis after starting ART; they did not have IRIS. Autopsy showed a mild-to-severe demyelinating leukoencephalopathy: HIV encephalitis (microglial giant cells and demonstrable HIV virus in microglial cells) and both perivascular and diffuse white matter CD8^+^ T-cell infiltration. These observations contrast with the usual pathology presentation of HIV encephalitis but without HIV-CD8E, in pathology cases seen before the introduction of ART (34). In our variant form, a subacute IRIS-related HIV-CD8E is superimposed upon HIVE. The patients described by Langford et al. (33) did not have acute demyelinating encephalomyelitis (ADEM) pathology, but neither did they have the acute-subacute clinical syndrome of HIV-CD8E.

Additionally, we describe a variant CD8E morphology: Table 1, #21 had a subacute IRIS-related HIV-CD8E superimposed upon HIVE with demonstrable virus on IHC; Table 1 #23 had HIV-CD8E arising in a well-controlled individual, with no risk event, and burnt-out HIVE, i.e., multinucleate giant cells, but no demonstrable virus on IHC, These represent active and inactive HIVE pathologies, respectively.

### No Risk Event Identified

HIV-CD8E can develop without warning or evident change in the patient's pVL and CD4 count—those with no risk event identified. We have identified this category more than other authors, but our critical review of the published cases shows this scenario is not limited to UK patients. A proportion of these show CSF viral escape, so undetermined events are occurring that affect the brain compartment immune balance and presumably the blood-brain barrier.

### ART-Naïve

The occurrence of HIV-CD8E in patients never treated with ART was unexpected. In three of the four patients so identified to date, their HIV infection was of many years duration, and their pVL high. Further, if HIV-CD8E occurs in untreated HIV, it is not clear why it was not described in the pre-ART era. Perhaps, as discussed above, it is a sampling artifact or due to its relative rarity at a time when classical HIVE CNS disease was common. Another possible explanation comes from the pathological study of early HIV infection; much of this is opportunistically derived from autopsies in asymptomatic persons performed for non-HIV/AIDS-related reasons [(35) and SBL personal observations]. Whilst few patients suffer a fulminating encephalopathy, the brains in the majority show similar features to HIV-CD8E: abundant CD8^+^ T-cells, few CD4^+^ T-cells, some astrocytosis and diffuse microgliosis (but no giant cells), and little or no demonstrable HIV antigen by IHC, although PCR analysis identified HIV-1 genome in a few. As such, we suggest that HIV-CD8E in untreated individuals could represent a delayed entrance of HIV virus into the brain or the delayed recognition of HIV antigens in the brain.

### EBV- Associated HIV-CD8E

A recent report from Ireland (26) described three patients with typical HIV-CD8E symptoms, two of whom had brain biopsies, showing the typical pathology, and all three survived. One of them had, by *in situ* hybridization, numerous EBV^+^ lymphocytes on biopsy, the other only very scanty cells. Two had low level EBV DNA in CSF, and serum EBV DNA was not detected.

EBV encephalitis is rarely described in adults with HIV (36). This raises the question: does EBV play a role in triggering HIV-CD8E or is it merely an innocent bystander? (26). Pathologists have long noted that in HIV^+^ biopsy and autopsy material from all non-brain tissues, occasional EBV^+^ lymphocytes are seen, and are generally ignored unless they are numerous or associated with a lymphoma.

EBV *in situ* hybridization was performed on many of the biopsies and autopsy samples in the UK case series and, where done, was negative (1, 13). Reviewing the non-Ireland global case series, most of the studies do not mention EBV. The large French series (7, 21) did analyses for EBV on blood and CSF and on the brain tissues: all cases were negative. Patients reported by Peluso et al. (5) had low level blood and negative CSF EBV DNA; additionally, other series documented negative blood EBV DNA (12, 15, 18, 19); and none of these studies looked for EBV in brain tissue.

So, it is possible that EBV infection, reactivated because of HIV, or acute, could play a role in the development of clinical HIV-CD8E, but its precise role is unclear. EBV *in situ* hybridization should be performed on all biopsy and autopsy brain samples where the syndrome is a diagnostic possibility; and a retrospective study on archival brain material would be informative.

### Tip of the Iceberg?

In an editorial accompanying the Lescure et al. article on HIV-CD8E (7) the authors suggest that the syndrome may be the severe “tip of the iceberg” of the range of HIV-associated cerebral pathology and resultant cognitive impairment (27). This supposes that there is a spectrum of brain pathology involving parenchymal CD8^+^ T-cell infiltration and microglial activation, with milder forms presenting as cognitive disorder (37) and more severe forms as HIV-CD8E and risk of death. Analysis of CSF in patients with mild cognitive impairment demonstrates raised activation markers (e.g., neopterin) (32, 38), so this hypothesis is plausible.

HIV-associated dementia has some but not exact pathological correlation with HIVE (39). Further studies of autopsy brain tissue, CSF and blood in HIV-infected patients with and without neurocognitive disorders show the complexity of the potential associations (40): brains from those with HIVE have much HIV RNA virus on PCR analysis, as expected, but in those with neurocognitive disorder and without HIVE, HIV RNA levels are similar to that in brains from those without neurocognitive disorder. The poorest neuropsychiatric scores, whilst correlating with brain HIV RNA, did not correlate with pre-mortem plasma and CSF viral loads. Overall, there is as yet insufficient evidence from systematically-acquired neuropathology of adults with HIV infection, both with and without cognitive impairment, to support the hypothesis of a continuous spectrum of clinical pathology from HIV infection through milder neurocognitive disorder, HIV-associated dementia, to death from HIV-CD8E (41, 42).

### Pathogenesis

There is a consensus that pathologically, HIV-CD8E differs and is distinct from classical HIVE, and is neither cerebral T-cell lymphoma or DILS (7). In DILS neuropathy, HIVp24 antigen is visible, whereas it is minimal or absent in brain tissue of those with HIV-CD8E.

In the UK case series, HIV p24 antigen was seen in only one brain (#21), and one other patient's brain (#1) contained HIV genome on PCR testing, indicating a latent infection. The association with CSF viral escape implies the presence of HIV in the CNS. It is recognized that HIV p24 immunohistochemistry has a practical threshold before HIV known to be present can be visualized (43), so negative IHC does not exclude the presence of HIV virus.

HIV-CD8E is not usually a destructive encephalitis with neuronophagia and significant white matter damage and vasculitis (as, for example, in multiple sclerosis and ADEM) (7). In only one reported HIV-CD8E patient is there an indication of significant histological brain damage (25).

The possibility that HIV-CD8E represents an ART-associated drug toxicity phenomenon is unlikely. The patients had experienced all types of ART medication available over the last two decades, with no consistent prescription pattern; similarly the CPE scores of the ART therapies encompassed the entire range (Table 1). Finally, several accounts note that once HIV-CD8E starts, it can become autonomous and catastrophic (implying an uncharacterised chaotic process), resulting in death unless clinicians instigate treatment.

### Disequilibrium Between HIV and Brain Immunity

Pathogenetic accounts in the literature include two broad explanations for HIV-CD8E.

Firstly, that it is a transient disequilibrium between HIV and brain immunity, which may or may not be triggered by HIV replication in the brain. Nearly all cases of HIV-CD8E have no detectable HIV virus by immunohistochemistry, although—as seen in UK patient #1—there is latent virus in the brain detectable by PCR techniques; and it is accepted that patients with HIV infection, even those well-controlled on ART, have latent virus in the brain when studied with highly sensitive techniques (44). In patients with treatment interruption, intercurrent infection and ART drug resistance, pVL are rising, and Table 3 confirms that, in some, the brain/CSF viral load is also increasing with viral escape; but in IRIS, the situation is reversed, with falling pVL, although there are insufficient data on brain/CSF viral load to be more confident. In the ART-naïve patients with HIV-CD8E, entry of HIV into the brain, or its recognition by the immune system, might be delayed.

In cases associated with intercurrent infection, there is suggested a novel introduction of HIV into the brain through an “immune distraction,” which allows CNS viral escape (11); additionally, intercurrent infection could, through cytokines, alter the blood brain barrier function, increasing its permeability.

CNS viral escape is a complex and incompletely understood phenomenon, and can be classified into asymptomatic, symptomatic and secondary (to intercurrent infection) (38). It is demonstrable (and explicable) in small numbers of patients with HIV-CD8E in the risk factor categories 1–3, but not with IRIS (Tables 1, 3). It can develop in patients with relatively high CD4^+^ T-cell counts, reflects activation of CNS infection, and may induce a CD8^+^ inflammatory response in some patients but not others—perhaps related to host genetic factors. More study of viral escape is needed, particularly in IRIS-related HIV-CD8E, but this is difficult given the brain swelling that occurs in the syndrome.

### CD4^+^/CD8^+^ T-Cell Imbalance and Microglial Activation

The second explanation is that HIV-CD8E is associated with a perturbation of the balance between CD4^+^ T-cells and CD8^+^ T-cells in the brain with recruitment of CD8^+^ T-cells (9, 15, 17, 18, 22, 25); this applies particularly to cases linked with IRIS (45). IRIS in HIV disease—which is associated with falling pVL, rising blood CD4^+^ and, usually, CD8^+^ T-cell levels—has no histopathological case definition, but it is accepted that increased numbers of CD8^+^ T-cells in tissues is characteristic (46).

Related to this, estimating by microscopy the CD4^+^ and CD8^+^ T-cell densities in brain tissue, those patients who have CD4^+^ cells present have a more favorable outcome, whereas those with no or minimal CD4^+^ T-cells are more likely to die (21). However, a CD4^+^/CD8^+^ T-cell imbalance within the brain cannot be the initiating process for HIV-CD8E since the brain parenchyma has no resident T-cells. It would be worthwhile re-examining more archived brain tissue material to explore the CD4^+^/CD8^+^ T-cell ratio and its possible prognostic significance.

It is agreed that B-lymphocytes play no role in HIV-CD8E, although in one IRIS-associated case (#9, Table 1), B-cells were prominent enough on biopsy to initially suggest a lymphoma.

A constant feature of HIV-CD8E (and other encephalitides unrelated to HIV infection) is activation of microglial cells. It is conceivable that this could have a primary pathogenic role, through chemokine and cytokine secretion changing the integrity of the blood brain barrier (see below) and providing chemo-attractants for T-cells to cross the barrier (47).

### Blood Brain Barrier

Thus, it is evident that there is no unifying detailed neuropathogenesis for HIV-CD8E beyond the general concept of changing and unbalanced cell immunity responses to HIV virus—which itself may be changing or static—in the brain. Regarding the critical blood brain barrier, whose tight junctions must open to let in the T-cells, there has been some research into its structure in man and animals in HIV infection, demonstrating molecular changes, but little on functional morphology (48). Its role in HIV-CD8E is unexplored, yet breakdown of this barrier must be a key process. The normal brain contains no histologically visible T-cells in the neuropil, only within the blood vessels. Usual histo-morphological observations in HIV-CD8E have not as yet shown abnormalities of the blood brain barrier structure at the endothelial level: this is a complex neurovascular unit composed of endothelial cells, their basement membrane in which pericytes are embedded, and foot processes of astrocytes on the outside (49). The observation of CD8^+^ T-cells passing through the vessel walls and accumulating around vessels do not inform about how they left the blood stream.

HIV-CD8E does not merely represent a non-specifically leaky blood brain barrier in the presence of increasing CD8^+^ T-cell counts in the blood: in the UK series, serial blood counts show declining numbers prior to the crisis (Figure 3). Pathogenetically there appears to be a process occurring in the CNS tissue compartment that is signaling, presumably directed at CNS HIV via cytokines, these T-cells to cross into the brain, and further study of the integrity of the neurovascular unit in HIV disease is needed (42).

### Corticosteroid Treatment

From the UK and global case data, 30% patients that received steroids died, by contrast with 69% that did not receive steroids. Potential mechanisms by which steroid therapy may be beneficial include: (a) closure of the tight junctions between endothelial cells at the blood brain barrier (49); (b) blocking of the activating effect of CD4-T-cell IL-2 on CD8-T-cells (17); (c) alteration of ligand expression on T-cells (e.g., L-selectin) and binding to endothelial cells (50); and (d) the T-lymphocyte killing effect of corticosteroids (51). These data may be subject to “survivor” bias, whereby patients presenting as “sudden death,” or in whom a diagnosis of HIV-CD8E is not considered, do not survive to receive corticosteroids whereas those living long enough to be investigated and who are diagnosed are much more likely to receive corticosteroids.

Whether changing the ART regimen at the at the same time as giving corticosteroids is also important in the management of diagnosed HIV-CD8E is unclear from both the UK series and from the global literature.

### A Future Scenario of HIV-CD8E?

Lastly, there is an eighth possible scenario for HIV-CD8E which, in the literature, does not appear to have yet happened. One approach toward a cure for HIV infection is to induce latently-infected cells [known to exist in the brain (44)] which do not express viral antigens, to restart expression, with the drug vorinostat (52). Then, in principle, ART could eliminate these HIV antigen-expressing cells when synergised with interventions to enhance HIV-cytolytic T-cell activity. Theoretically, HIV-CD8E could develop in those treated with this “kick and kill” regime; initial trials of this treatment strategy have not to date reported serious adverse effects.

## Conclusion

The 53 cases reported and reviewed here comprise the largest yet number of HIV-associated CD8 encephalitis. The clinical phenotype is a person with HIV presenting with symptoms and signs related to marked cerebral inflammation and swelling. The pathology is of supra- and sub-tentorial brain involvement, with variable swelling; histopathologically, there is diffuse white matter parenchymal infiltration by CD8^+^ T-cells with little or no CD4^+^ T-cell or B-cell involvement, and microglial activation; gray matter is much less involved and there is no neuronophagia. HIV-CD8E is distinguished from other HIV-related brain clinical pathologies: it is not HIVE, T-cell lymphoma, PRES (posterior reversible encephalopathy syndrome (25), HIV vasculitis (53), or other vasculitic brain disease, ADEM, an opportunistic infection, or classical virus-associated or autoimmune encephalitis.

Black African ethnicity is an important risk factor, suggesting a genetic contribution to pathogenesis of HIV-CD8E. Of the risk factors precipitating the syndrome, the most important are interruption of anti-retroviral treatment, and IRIS after commencement of ART. Intercurrent infection and ART drug resistance contribute, but the syndrome also occurs for unexplained reasons in well-controlled patients, and in a few who have never taken ART. Pathogenetically, there is evidently an acute imbalance between the host brain vs. blood compartments in terms of HIV load and cellular immune response, that permits the blood brain barrier to become leaky and allow ingress of vast numbers of CD8^+^ T-cells into the brain. The phenomenon of CNS viral escape is a background immunological phenomenon across all the risk factor types with the exception of IRIS, but there have been insufficient patients studied with CSF vs. plasma viral load data to draw more conclusions. At present no single pathogenetic mechanism accounts for all cases and risk factors for HIV-CD8E. The importance of considering the diagnosis in HIV patients whose brain function unexpectedly deteriorates, and rapidly excluding as far as possible alternative diagnoses is evident: cortico-steroid therapy can save the lives of patients with HIV-CD8E.

Future studies of HIV-CD8E pathogenesis can include: close correlation of HIV clade with disease; collecting CSF at autopsy (it being difficult in life due to brain swelling); using unfixed brain material to study microglial cells' secretions, chemokines and cytokines; using polymorphism analysis to pursue the ethnic predisposition to HIV-CD8E; comparing HIV-1 env sequences isolated from CNS and extra-CNS cells, to evaluate a possible change in HIV co-receptor tropism in HIV-CD8E; consideration of changing ART regimen inpatients diagnosed in life.

## Data Availability Statement

The original contributions presented in the study are included in the article/Supplementary Material, further inquiries can be directed to the corresponding author/s.

## Ethics Statement

Ethical review and approval was not required for the study on human participants in accordance with the local legislation and institutional requirements. Written informed consent for participation was not required for this study in accordance with the national legislation and the institutional requirements.

## Author Contributions

SL described the first cases of HIV-CD8 encephalitis in 2004 (1), performed pathological studies, co-wrote the first draft of the manuscript, and co-wrote subsequent drafts and the final draft of the manuscript. RM provided direct clinical care for three of the patients in this report, described the first cases of HIV-CD8 encephalitis in 2004 (1), co-authored a subsequent case report of HIV-CD8 encephalitis in 2017, performed the literature review, contacted the authors of previously published reports of HIV-CD8 encephalitis in order to ascertain correct assignment of “risk event” where uncertainty existed, co-wrote the first draft of the manuscript, and co-wrote subsequent drafts and the final draft of the manuscript. SN repeated and confirmed the accuracy of the literature review done by RM, commented critically on the first draft, and co-wrote subsequent drafts and the final draft of the manuscript, provided expert knowledge, understanding and critical interpretation of data about this condition from a LMIC perspective. KW performed histopathological studies, co-wrote the first draft of the manuscript, took many of the histopathology photographs, and commented critically on subsequent drafts, as well as the final draft of the manuscript, and provided critical interpretation of data about this condition from a LMIC perspective. All collaborators have approved the final submitted version of the manuscript.

## Conflict of Interest

The authors declare that the research was conducted in the absence of any commercial or financial relationships that could be construed as a potential conflict of interest. The reviewer GM declared a shared affiliation, with no collaboration, with one of the authors SN to the handling Editor.

## Glossary

**ADEM**, Acute disseminated encephalomyelitis

**ART**, antiretroviral therapy

**CNS**, Central nervous system

**CPE**, Central nervous system penetration effectiveness (CPE) score provides an indication of each antiretroviral drug's capacity to penetrate the central nervous system and inhibit HIV replication. The CPE score is based on pharmacokinetic, pharmacodynamic, and physio-chemical properties of each drug. A high CPE score has been associated with a reduction in cerebrospinal fluid HIV-1 RNA (54)

**CSF**, Cerebrospinal fluid

**DILS**, Diffuse infiltrative lymphocytosis syndrome

**EBV**, Epstein-Barr virus

**EBER**, Epstein-Barr virus-encoded small RNAs

**HIV-CD8E**, HIV-associated CD8 encephalitis (HIV-CD8E) is a severe inflammatory disorder dominated by infiltration of the brain by CD8^+^ T-lymphocytes. It occurs in people with HIV, typically when the virus is apparently well-controlled by antiretroviral treatment. HIV-CD8E presents with symptoms and signs related to marked cerebral inflammation and swelling, and can lead to coma and death unless treated promptly with corticosteroids. Risk events such as intercurrent infection, antiretroviral therapy interruption, immune reconstitution inflammatory syndrome after starting ART, and concomitant associations such as cerebrospinal fluid HIV viral escape have been identified. HIV-CD8E has characteristic brain histopathology: diffuse cerebral infiltration by CD8^+^ T-cells and microglial activation, and the absence of other specific causes of encephalitis (11)

**HIVAN**, HIV-associated nephropathy

**HIVE**, HIV encephalitis (HIVE) is due to productive infection in the CNS by HIV and affects predominantly the white matter, basal ganglia and brain stem. Pathologically lesions are diffuse or multifocal and are characterized by abundant stainable HIV, reactive astrocytosis and intense microglial activation with frequent multi-nucleated giant cells. It presents clinically with a sub-acute to chronic subcortical dementia syndrome (55)

**HIV p24**, The gag protein p24 is a component of the HIV capsid the epitope for antibody innumohistochemistry staining of HIV virus

**IHC**, immunohistochemistry

**IRIS**, Immune reconstitution inflammatory syndrome (IRIS) is a collection of inflammatory disorders associated with clinical or radiological worsening of previously diagnosed or sub-clinical infections following initiation of ART. Typically presentation occurs within 3 months of initiation of ART and is associated with a >1log_10_ reduction in plasma HIV viral load compared to baseline (15)

**PCR**, polymerase chain reaction

**pVL**, The plasma HIV-1 RNA viral load (pVL) is a measure of the number of HIV-1 RNA copies/ml of blood

**PRES**, Posterior reversible encephalopathy syndrome, associated with transient vasoconstriction which is not vasculitic

**VARIANT HIV-CD8E**, a pathological scenario of global CD8^+^ T-cell infiltration but with a background of HIVE lesions in the white matter

these are either active (demonstrable virus with HIVp24 IHC) or inactive (multinucleate giant cells but no stainable virus)

**VIRAL ESCAPE**, in patients receiving antiretroviral therapy viral escape is defined by detectable HIV-1 RNA in cerebrospinal fluid despite an undetectable or lower than cerebrospinal fluid level in plasma (6).
