# Supplementary material for: HIV-Associated CD8 Encephalitis: A UK Case Series and Review of Histopathologically Confirmed Cases
Source: Front Neurol. 2021 Apr 1;12:628296. doi: 10.3389/fneur.2021.628296 (PMC8047670; doi:10.3389/fneur.2021.628296)
Supplement: Supplementary file 1 [file Table_1.docx]

Supplementary Table. Previously published HIV-CD8E cases without histopathology; censored on 02 November 2020

| Year of  report | Country of report | Patient  ethnicity | Outcome | Risk event category | CSF viral escape | Cortico-steroids  given | Internal case ref | Reference |
| --- | --- | --- | --- | --- | --- | --- | --- | --- |
| 2009 | Germany | Caucasian | Alive | 4 | ND | Yes | #1 | (1) |
|  |  | Caucasian | Alive | 4 | ND | Yes | #2 |  |
|  |  | Caucasian | Alive | 4 | ND | yes | #3 |  |
|  |  |  |  |  |  |  |  |  |
|  |  |  |  |  |  |  |  |  |
| 2009 | Canada | Aboriginal | Alive | 4 | ND | No | #1 | (2) |
|  |  | Caucasian | Alive | 4 | ND | No | #6 |  |
|  |  | Caucasian | Alive | 4 | ND | No | #7 |  |
|  |  |  |  |  |  |  |  |  |
| 2013 | France | African and Caucasian patients, but without individual ethnicity data presented | Alive  Died  Died  Alive | 2  NA  2  NA | No  ND  Yes  Yes | Yes  Yes  Yes  Yes | #11  #12  #13  #14 | (3) (cases 11-14)  (4) (case 6a) |
|  |  |  |  |  |  |  |  |  |
| 2016 | UK | NA | Alive | 3 | Yes | Yes |  | (5) |
|  |  |  |  |  |  |  |  |  |
| 2019 | India | Indian | Alive | 1 | Yes | Yes |  | (6) |
|  |  |  |  |  |  |  |  |  |
| 2020 | Ireland | Black African | Alive | 5 | Yes | Yes | #2 | (7) |

Key: Risk category of CD8E: 1 = well controlled HIV infection, no trigger identified; 2 = intercurrent infection or Hodgkin lymphoma;

3 = treatment interruption; 4 = IRIS on ART; 5 = ART drug resistance; 6 = not on ART. ND = CSF viral load not measured. NA = not available

REFERENCES

1. Ringelstein, A., Oelschlaeger, C., Saleh, A., Mathys, C., Dziewasm, R., Niederstadtm, T., et al. (2012). Severe aseptic leucoencephalopathy as immune reconstitution inflammatory syndrome in caucasian and African patients. AIDS. 23, 1435-7.
2. McCombe, J.A., Auer, R.N., Maingat, F.G., Houston, S., Gill, M.J., Power, C. (2009). Neurologic immune reconstitution inflammatory syndrome in HIV/AIDS: outcome and eoidemiology. Neurol.72, 835-841.
3. Lescure, F.-X., Moulignier, A., Savatovsky, J., Amiel, C., Carcelain, G., Molina, J.M., et al. (2013). CD8 encephalitis in HIV-infected patients receiving cART: a treatable entity. Clin. Infect. Dis. 57, 101-108.
4. Gray, F., Lescure, F.-X., Adle-Biassette, H., Polivka, M,. Gallien, S., Pialoux, G., et al. (2013). Encephalitis with infiltration by CD8+ lymphocytes in HIV patients receiving combination antiretroviral therapy. Brain. Pathol. 23, 525-533.
5. Salam, S., Mihalova, T., Ustianowski, A., McKee, D., Siripurapu, R. (2016). Relapsing CD8+ encephalitis – looking for a solution. BMJ. Case. Rep. bcr-2016-214961.
6. Mirgh, S.P., Mishra, V.A., Harbada, R.K., Sorabjee, J.S. (2019). Knowing the unknown – CD8 encephalitis: a novel form of HIV-associated neurocognitive disorder. Neurol. India. 67, 261-264.
7. Kerr, C., Adle-Biassette, H., Moloney, P.P. et al. (2020). CD8 encephalitis with CSF EBV viraemia and HIV drug resistance, a case series. Brain. Behaviour. Immunity. – Health. 9, 100164.
